# Altered Expression of Ganglioside Metabolizing Enzymes Results in GM3 Ganglioside Accumulation in Cerebellar Cells of a Mouse Model of Juvenile Neuronal Ceroid Lipofuscinosis

**DOI:** 10.3390/ijms19020625

**Published:** 2018-02-22

**Authors:** Aleksandra Somogyi, Anton Petcherski, Benedikt Beckert, Mylene Huebecker, David A. Priestman, Antje Banning, Susan L. Cotman, Frances M. Platt, Mika O. Ruonala, Ritva Tikkanen

**Affiliations:** 1 Center for Membrane Proteomics, Goethe University of Frankfurt, 60438 Frankfurt am Main, Germany; aleksandra.somogyi@ucc.ie (A.S.); apetcherski@mednet.ucla.edu (A.P.); 2 Institute of Biochemistry, Medical Faculty, University of Giessen, Friedrichstrasse 24, 35292 Giessen, Germany; Benedikt.Beckert@biochemie.med.uni-giessen.de (B.B.); Antje.banning@biochemie.med.uni-giessen.de (A.B.); 3 Department of Pharmacology, University of Oxford, Mansfield Road, Oxford OX1 3QT, UK; mylene.huebecker@pharm.ox.ac.uk (M.H.); david.priestman@pharm.ox.ac.uk (D.A.P.); frances.platt@pharm.ox.ac.uk (F.M.P.); 4 Center for Genomic Medicine, Department of Neurology, Massachusetts General Hospital Research Institute, Harvard Medical School, 185 Cambridge Street, Boston, MA 02114, USA; cotman@helix.mgh.harvard.edu

**Keywords:** Batten disease, neuronal ceroid lipofuscinosis, CLN3, lysosomal storage disorders, glycosphingolipids, gangliosides

## Abstract

Juvenile neuronal ceroid lipofuscinosis (JNCL) is caused by mutations in the *CLN3* gene. Most JNCL patients exhibit a 1.02 kb genomic deletion removing exons 7 and 8 of this gene, which results in a truncated CLN3 protein carrying an aberrant C-terminus. A genetically accurate mouse model (*Cln3^Δex7/8^* mice) for this deletion has been generated. Using cerebellar precursor cell lines generated from wildtype and *Cln3^Δex7/8^* mice, we have here analyzed the consequences of the *CLN3* deletion on levels of cellular gangliosides, particularly GM3, GM2, GM1a and GD1a. The levels of GM1a and GD1a were found to be significantly reduced by both biochemical and cytochemical methods. However, quantitative high-performance liquid chromatography analysis revealed a highly significant increase in GM3, suggesting a metabolic blockade in the conversion of GM3 to more complex gangliosides. Quantitative real-time PCR analysis revealed a significant reduction in the transcripts of the interconverting enzymes, especially of β-1,4-*N*-acetyl-galactosaminyl transferase 1 (GM2 synthase), which is the enzyme converting GM3 to GM2. Thus, our data suggest that the complex a-series gangliosides are reduced in *Cln3^Δex7/8^* mouse cerebellar precursor cells due to impaired transcription of the genes responsible for their synthesis.

## 1. Introduction

Juvenile neuronal ceroid lipofuscinosis (JNCL) is a lysosomal storage disorder caused by mutations in the *CLN3* gene. Most JNCL patients worldwide are homo- or heterozygous for a deletion that removes exons 7 and 8 together with the bridging intron. A genetically authentic mouse model for this mutation has been generated, and it has been shown to recapitulate many of the pathogenic features of human JNCL disease [1,2,3,4,5,6]. One of the first symptoms in JNCL patients is visual system impairment that frequently manifests at the age of 5—8 years and leads to blindness due to retinal degeneration. Within the first decade of life, the patients’ mental and physical capabilities continue to decline, and epileptic seizures become evident. In addition to the central nervous system, JNCL patients also exhibit pathologic features in the cardiac and immune systems. Towards the end of the second decade of life, many patients rapidly deteriorate and become wheelchair bound, with death usually occurring by the age of 25.

The gene product, CLN3 protein, is a transmembrane protein that is predicted to span the membrane 6 times, with both N- and C-terminus residing in the cytosol [7,8]. Although the exact molecular function of the CLN3 protein has not been conclusively verified, it has been linked to various cellular processes such as autophagy, lysosomal pH, vesicular trafficking and cation homeostasis in lysosomes [2,4,9,10]. Furthermore, the CLN3 protein has been shown to be associated with cell proliferation, control of cell cycle and apoptosis. However, it is not clear if these phenotypic features are directly caused by an impairment of CLN3 protein function or if they represent secondary effects.

**Figure 1:**
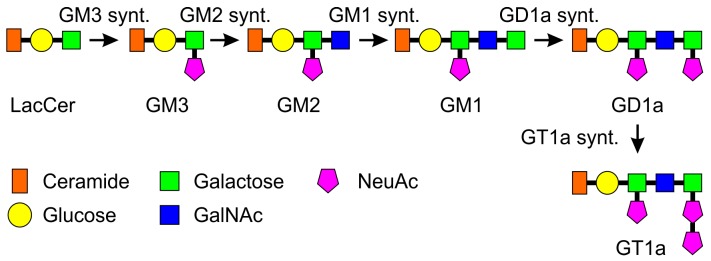
Synthesis and structures of a-series gangliosides. Shown are the synthesis steps and structures of the a-series gangliosides, starting from LacCer. Successive addition of glycosyl residues by glycosyltransferases results in synthesis of the complex gangliosides from GM3. Please see Introduction for details. Synt. = synthase.

Glycosphingolipids (GSLs) are important glycan structures mainly residing at the cell surface, especially in neuronal cells. GSLs comprise ceramide linked to a varying number of sugar residues. Gangliosides are a subgroup of GSLs, distinguished by the presence of sialic acid residues, mainly N-acetylneuraminic acid in humans [11,12]. The biosynthesis pathway and the structures of the a-series gangliosides with their synthesizing enzymes are shown in Figure 1, and the genes and the respective enzymes are summarized in Table 1. GM3 is synthesized from lactosylceramide (LacCer) by the enzyme LacCer sialyltransferase (GM3 synthase) and is the precursor for the a-series gangliosides. Conversion to GM2 takes place by the action of GM2 synthase (GalNAc transferase). Addition of a galactose residue to GM2 by galactosyltransferase 2 (GM1 synthase) results in GM1a ganglioside, which can further be converted into GD1a upon terminal sialylation by a sialyltransferase (GD1a synthase). GT1a can be synthesized from GD1a by sialylation by GT1a synthase. The same enzymes are responsible for the synthesis of the b- and c-series gangliosides where the synthesis precursors arise from GM3 by further sialylation. For a detailed description of the structures of the b- and c-series, please refer to the review by Daniotti and Iglesias-Bartolomé [13]. Ganglioside synthesis takes place mainly in the Golgi where glycosyltransferases sequentially add sugar residues to the lipids. Glycosyltransferase expression is regulated especially at the transcriptional level, but also by formation of protein complexes comprising several glycosyltransferases [14,15].

In the brain, gangliosides are especially abundant and important for the development and maintenance of the organ. In particular, a-series gangliosides have been shown to be essential for neurite outgrowth and cell survival [16,17]. The special role of gangliosides in the central nervous system is also highlighted by severe human diseases, the gangliosidoses, which result from genetic defects mainly in the genes involved in the degradation of gangliosides. For example, Tay-Sachs and Sandhoff diseases, collectively known as the GM2 gangliosidoses, result from impaired degradation of GM2 ganglioside (For review, see [11]). In addition, secondary accumulation of gangliosides is observed in sphingolipid storage disorders such as Niemann-Pick Type C disease, in which the mutated genes are not directly involved in ganglioside metabolism. On the other hand, only few human diseases have been confirmed to result from genetic defects in the ganglioside biosynthesis pathways [18,19,20]. However, diseases that impair sphingolipid metabolism collectively show that accumulation of specific gangliosides is highly deleterious especially to the central nervous system.

Previous studies have shown that in various NCLs, accumulation of gangliosides or their precursors may occur. In JNCL cells, accumulation of ceramide has been described [21], and CLN3 protein has been proposed to be involved in galactosylceramide transport [22,23]. Here, we have analyzed the levels of the more complex gangliosides, with special focus on the a-series, in cerebellar granule cell precursor lines derived from homozygous *Cln3^Δex7/8^* mice. We show that specific alterations in the levels of ganglioside expression are present in this JNCL cell model. In particular, an accumulation of GM3 ganglioside was observed, whereas levels of the more complex gangliosides, especially GM1a, were reduced. Furthermore, we detected significantly reduced transcripts of many of the genes involved in ganglioside biosynthesis and degradation, implicating that in JNCL, there may be secondary defects that result in altered ganglioside levels.

## 2. Results

**Figure 2:**
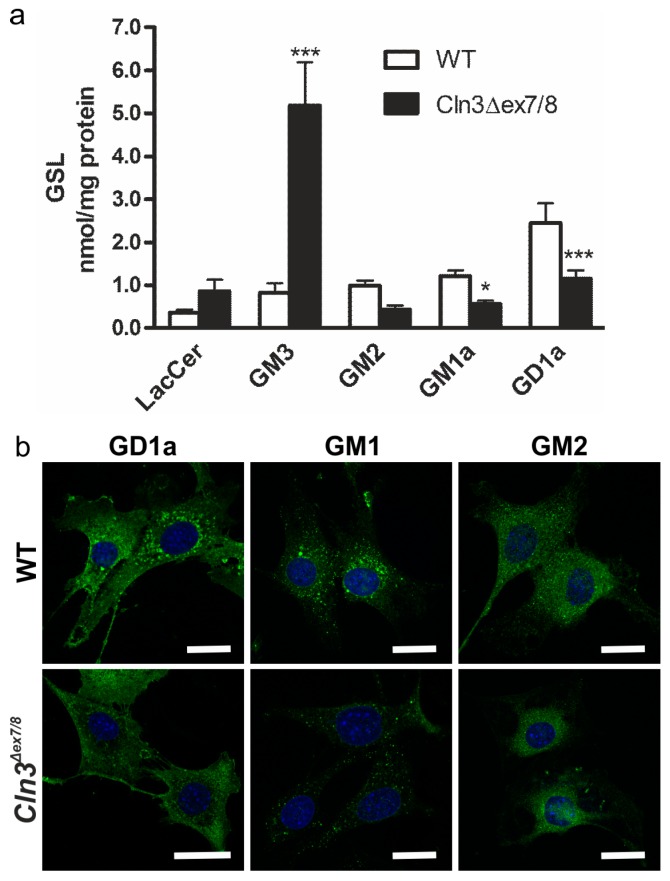
Alterations in gangliosides in the cerebellar precursor cells from the *Cln3^Δex7/8^* mouse model. (**a**) Quantitative HPLC analysis of glycosphingolipids in wildtype (WT) and *Cln3^Δex7/8^* cells shows increased levels of GM3, together with a reduction in GM1a and GD1a levels. Statistical analysis with 2-way ANOVA. *p* values * *p* < 0.05; *** *p* < 0.001; (**b**) Staining of GD1a, GM1a and GM2 in WT and *Cln3^Δex7/8^* cells. GM1a and GD1a levels are reduced in ***C****ln3^Δex7/8^* cells as compared with WT cells. The cells were immunostained with specific antibodies against the respective gangliosides and detected with a secondary antibody coupled to Alexa 488. Blue staining shows the nuclei stained with DAPI. Scale bar: 20 µm.

Intracellular accumulation of gangliosides is associated with several lysosomal storage disorders. In this study, we have quantitatively analyzed the a-series gangliosides in the mouse cerebellar granule neuronal precursor cell lines generated from wildtype (WT) and homozygous *Cln3^Δex7/8^* mice [2]. Lipids were extracted from cultured cells and analyzed by high performance liquid chromatography (HPLC, Figure 2a). Our data revealed a highly significant increase in the ganglioside GM3 in *Cln3^Δex7/8^* cells (*p* < 0.001). Furthermore, GM1a and GD1a gangliosides were significantly decreased in *Cln3^Δex7/8^* cells (*p* < 0.05 and *p* < 0.001, respectively), as compared with WT cells. A tendency for increased LacCer (*p* = 0.126) and decreased GM2 (*p* = 0.075) was also observed, but these data were not significant. We were unable to detect the b-series gangliosides, including GD3, by HPLC analysis, indicating that the levels of these gangliosides are extremely low in these cells. In agreement with the HPLC data, immunofluorescence staining of *Cln3^Δex7/8^* cells showed a reduced amount of GD1a and GM1a, whereas GM2 was not considerably altered (Figure 2b). Unfortunately, specific, validated antibodies for GM3 are not commercially available that would allow its detection by immunofluorescence.

**Figure 3:**
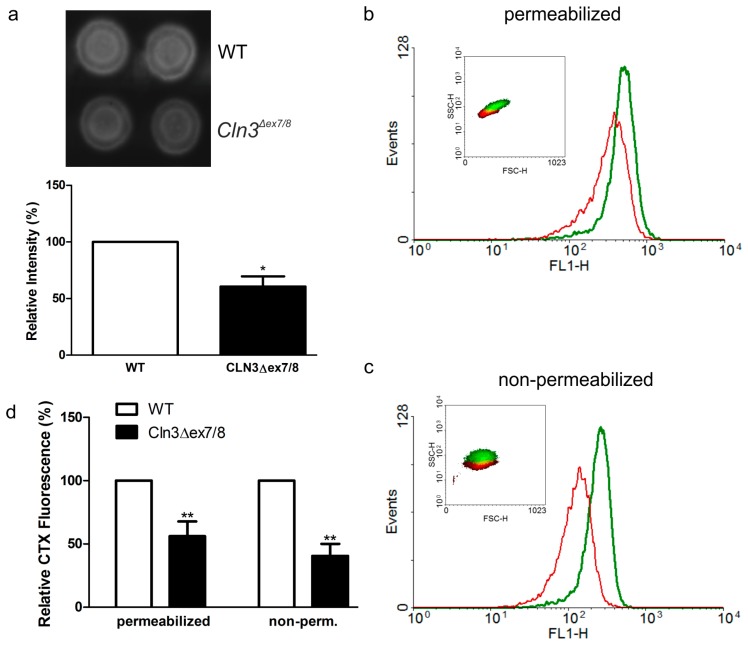
Reduced binding of cholera toxin on *Cln3^Δex7/8^* cells by dot blot and FACS analysis. (**a**) Dot blot of cholera toxin (CTX) binding to WT or *Cln3^Δex7/8^* cell lysates. Equal amounts of total protein from cell lysates were dot blotted onto nitrocellulose membrane that was incubated with HRP-coupled CTX. The signal was detected using enhanced chemiluminescence and quantified. Representative fluorescence intensity profiles of (**b**) permeabilized or (**c**) non-permeabilized WT (green) and *Cln3^Δex7/8^*(red) cells. The cells were incubated with FITC-coupled CTX to detect surface and intracellular GM1a pools and analyzed by flow cytometry. Insets in (**b**,**c**) represent associated forward and side-scatter plots to demonstrate size distributions of WT (green) and *Cln3^Δex7/8^* cells (red). (**d**) Quantification of the flow cytometry data from four independent experiments shows a significant reduction of CTX-FITC binding in both permeabilized and non-permeabilized *Cln3^Δex7/8^* cells. Significance in (**a**,**d**) was assessed using Student’s *t* test. * *p* < 0.05; ** *p* < 0.01.

GM1a ganglioside also functions as a high-affinity receptor for the cholera toxin B subunit (CTX). Thus, CTX binding to *Cln3^Δex7/8^* cells was used to quantitatively assess GM1a levels. Dot blot analysis with horseradish peroxidase (HRP) coupled CTX (Figure 3a) revealed a significantly reduced CTX binding to *Cln3^Δex7/8^* cell lysates. To study if the reduction of GM1a fraction was global or restricted to the plasma membrane or intracellular compartments, flow cytometry analysis of bound CTX-FITC was performed in digitonin-permeabilized (Figure 3b, plasma membrane and intracellular pools) and non-permeabilized cells (Figure 3c, plasma membrane pool). A significantly reduced CTX-FITC binding was detected in both permeabilized and non-permeabilized *Cln3^Δex7/8^* cells (Figure 3d), but the degree of reduction in non-permeabilized cells was slightly higher than in permeabilized cells.

**Figure 4:**
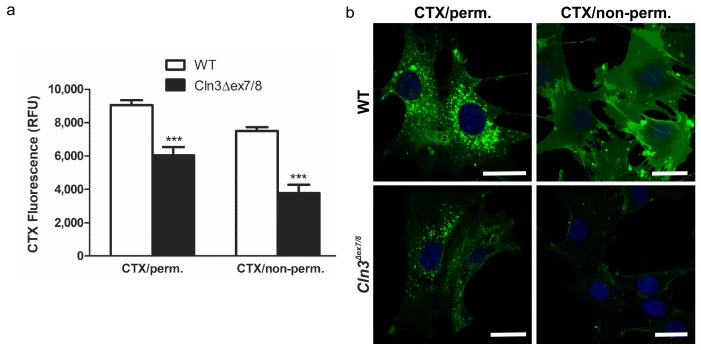
Reduced binding of cholera toxin on permeabilized and non-permeabilized *Cln3^Δex7/8^* cells. (**a**) Permeabilized or non-permeabilized WT and *Cln3^Δex7/8^* cells were incubated with FITC-coupled CTX and the fluorescence was quantitatively measured using a plate reader. Significance was assessed from three independent experiments using 2-way Anova. *** *p* < 0.001. (**b**) Permeabilized or non-permeabilized WT and *Cln3^Δex7/8^* cells were stained with A488-coupled CTX and imaged using a confocal fluorescence microscope. Blue staining shows the nuclei stained with DAPI. Scale bar: 20 µm. Data are representative for 3 independent experiments.

To verify the flow cytometry data, the binding of CTX-FITC to permeabilized or non-permeabilized WT and *Cln3^Δex7/8^* cells was quantified using a fluorescence plate reader (Figure 4a). A significant difference in CTX intensity between WT and *Cln3^Δex7/8^* cells was measured both with and without permeabilization. Again, the degree of reduction in CTX-FITC staining was higher in non-permeabilized *Cln3^Δex7/8^* cells. These data imply that not only the amount but also the relative distribution of GM1a might be altered in *Cln3^Δex7/8^* cells. Fluorescent staining of the cells with CTX-A488 (Figure 4b) also showed a clear reduction of CTX staining in both permeabilized and non-permeabilized *Cln3^Δex7/8^* cells. Taken together, the above data demonstrate that GM1a ganglioside amount and cellular distribution may be altered in *Cln3^Δex7/8^* cells.

**Figure 5:**
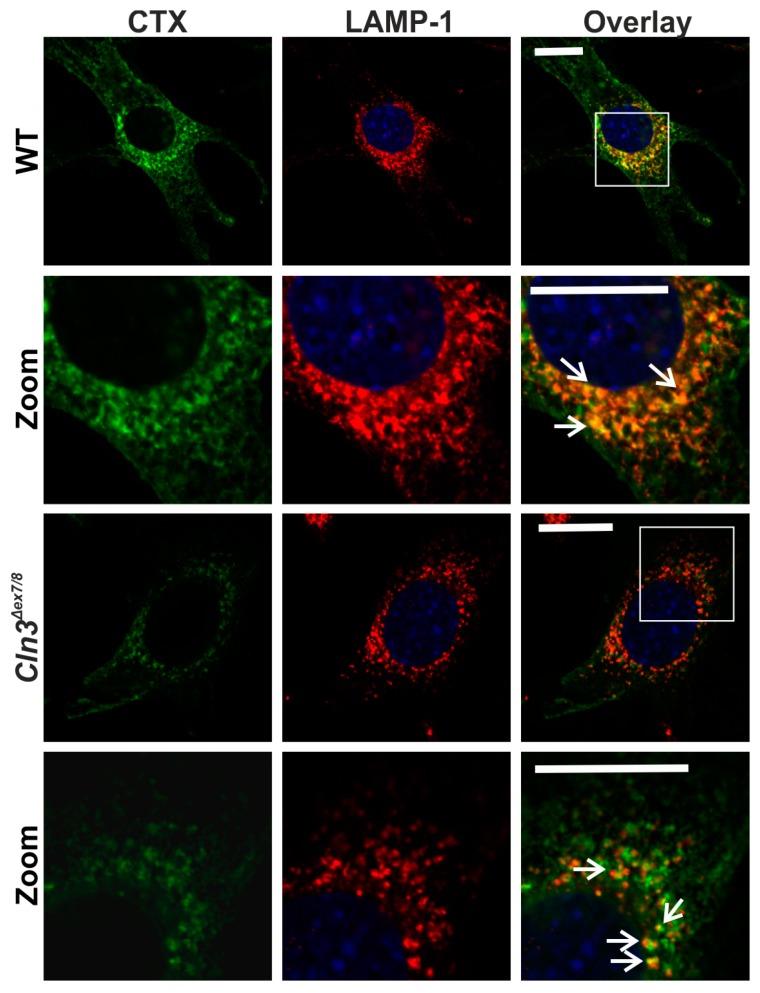
GM1a partially colocalizes with lysosomes in both WT and *Cln3^Δex7/8^* cells. The cells were stained with CTX-A488 (green) and anti-LAMP1 antibody (red) and imaged using a confocal microscope. Second and bottom row show an enlargement of the perinuclear area, depicted by a white frame in the overlay images on the first and third rows, respectively. Arrows point to examples of colocalization. The green signal in the overlay image of the *Cln3^Δex7/8^* cells was enhanced using the “best fit” function of the Zeiss Zen software to better depict the colocalization. Scale bar: 20 µm. Images show representative data from 3 independent experiments.

Since JNCL is a lysosomal storage disorder with alterations in endosomal/lysosomal system, the localization of the intracellular CTX in lysosomes was studied using staining with anti-LAMP-1 antibodies in CTX-A488 labeled WT and *Cln3^Δex7/8^* cells (Figure 5). In both cell lines, the CTX staining was partially colocalized with the LAMP-1 staining, indicating that although the *Cln3^Δex7/8^* cells exhibit less CTX staining, the intracellular portion was still mainly localized in the late endosomal/lysosomal compartments. 

The cellular levels of gangliosides may reflect changes either in their biosynthesis or degradation. To check if the expression of the enzymes responsible for the synthesis and degradation of a-series gangliosides was altered, quantitative real-time PCR experiments were performed (Table 2). The role of these proteins in ganglioside synthesis/degradation is shown in Section 3. Interestingly, the transcript levels of many of the genes encoding enzymes related to ganglioside metabolism were either significantly reduced (*B4GalNT1, HexA* and *Neu1*) or showed a tendency to be reduced (*Glb1*). Furthermore, cathepsin A/protective protein gene transcript (*Ctsa*) was significantly reduced, whereas the transcript of GM2 activator protein (*Gm2a*) was not changed. The GM1a sialyltransferase gene (*St3gal2*) transcription was not altered. Importantly, the *B3GalT4* gene for the enzyme converting GM2 to GM1a was approximately 4.5-fold upregulated, which might represent an attempt of the cells to compensate for the reduced GM1a level. These data suggest that in the *Cln3^Δex7/8^* cerebellar precursor cells, the synthesis and degradation of the a-series gangliosides may be dysregulated due to changes in the expression of the respective enzymes.

**Figure 6:**
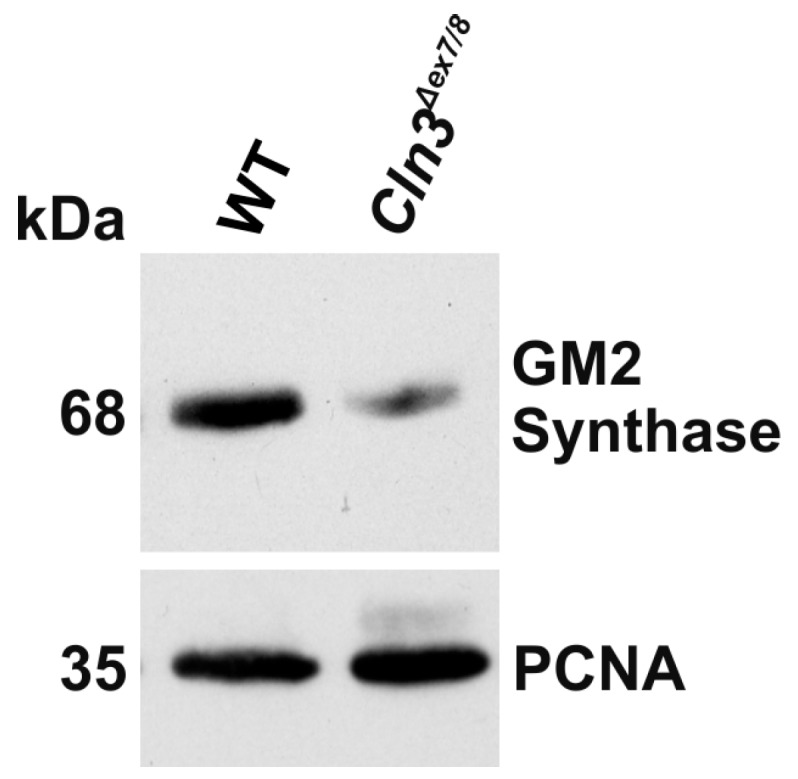
Reduced expression of GM2 synthase in *Cln3^Δex7/8^* cells. Cell lysates of WT and *Cln3^Δex7/8^* cells were analyzed by Western blot using an antibody specific for the GM2 synthase. PCNA (proliferating cell nuclear antigen) was used as a loading control. Data are representative for three independent experiments.

Since the transcript level of GM2 synthase (*B4galNT1* gene) was highly significantly reduced, we tested by Western blot if the protein amount of this enzyme was also altered (Figure 6). Indeed, we observed a significant reduction of GM2 synthase protein in the *Cln3^Δex7/8^* (33 ± 9.9%) vs. WT (100% ± 11%) cells (*t* test, *p* = 0.026). This would be consistent with the observed accumulation of GM3 and reduced transcript amount of this enzyme.

## 3. Discussion

We have here shown that the levels of the a-series gangliosides are altered in the cerebellar precursor cell line derived from the genetically accurate *Cln3^Δex7/8^* mouse model for JNCL. The most striking features were the reduction of GM1a and GD1a ganglioside levels and the concomitant increase in GM3. These data are in accordance with our studies using the Multi-Epitope-Ligand-Cartography [24] in brain slices of homozygous *Cln3^Δex7/8^* mice, where we also observed a reduced binding of CTX [25].

Previous studies have provided evidence for altered levels of glycosphingolipids and their precursors in JNCL cells. The Boustany group has shown that in JNCL patient fibroblasts homozygous for the major *CLN3* deletion mutation, the levels of ceramide, sphingomyelin, galactosylceramide (GalCer) and glucosylceramide were increased [22]. In studies with JNCL lymphoblasts with the same *CLN3* genotype, similar alterations were detected, together with specific changes in the cellular localization of the early GSL synthesis intermediates [23]. For example, GalCer was found to accumulate in the ER and Golgi fractions of the membranes in JNCL cells. This is consistent with data showing that the WT CLN3 protein exhibits a binding domain for GalCer and might thus be involved in GalCer transport towards the periphery [22]. Although CLN3 has been suggested to be a mainly endosomal/lysosomal protein, accurate detection of the localization of the endogenous protein is a major problem, since the antibodies available are not able to recognize the endogenous protein and many of them appear to be unspecific [26]. Overexpression studies, however, have revealed a mainly endosomal/lysosomal localization of CLN3 (see e.g., [7,27,28]). However, ganglioside synthesis takes place mainly in the early secretory route, and CLN3 trespasses these compartments on its way towards lysosomes. Thus, it is possible that CLN3 *en route* interacts with the ganglioside precursors or even the enzymes responsible for their synthesis. Another possibility is that malfunction of CLN3 protein exhibits an impact on signaling processes, which in turn may result in impaired transcription of ganglioside synthesis and degradation genes. The molecular mechanisms of how impairment of CLN3 function affects ganglioside synthesis should be addressed in further studies.

Although the abundance of the early GSL synthesis intermediates in JNCL has been addressed in several studies (see above), very little data are available on levels of the more complex sphingolipids. Kang et al. have shown that GM3, GM2 and GM1a are modestly increased in JNCL lymphoblasts, whereas sphingomyelin was found to be reduced [29]. Our data are partly contradictory to these findings, as we observed a significant increase in GM3 and a significant decrease in GM1a and GD1a. However, these differences might be due to the different cell systems used (patient lymphoblasts vs. mouse cerebellar precursor cell lines) or the methods that were used for GSL detection. Whereas Kang et al. used thin-layer chromatography [29], our approach is based on the highly sensitive and quantitative HPLC method. Furthermore, we have normalized our data to total protein amount, whereas Kang et al. normalized to the ganglioside GT1b of the b-series, which is not expressed in the cells used in our study [29].

**Figure 7:**
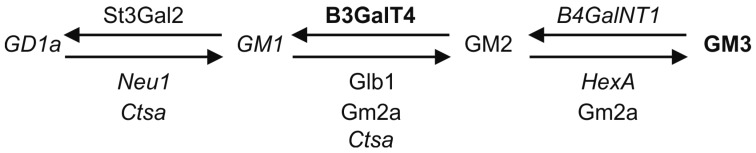
Summary of the alterations in gangliosides and their modifying genes in *Cln3^Δex7/8^* cells. Molecules found to be increased are shown in bold, those reduced in italics. Standard font depicts no change. Please see Discussion for details.

Since JNCL is a lysosomal storage disorder, the impaired lysosomal function might be expected to affect the lysosomal degradation of GSLs and cause an accumulation of the more complex gangliosides. However, we only observed an accumulation of GM3, whereas the more complex gangliosides were reduced. Our qPCR data show a profound dysregulation of the gene network involved in the synthesis and degradation of a-series gangliosides. Figure 7 summarizes the interconversion pathways of a-series gangliosides and the genes required for their synthesis (above the arrow) and degradation (below the arrows) and shows a summary of the qPCR data (genes reduced are shown italics, genes increased in bold). Especially intriguing is the highly significant reduction of the mRNA and protein level of the GM2 synthase enzyme (*B4GalNT1* gene) which converts GM3 to GM2. In the absence of this enzyme, one would expect to see an accumulation of GM3 and reduction in the levels of the more complex a-series gangliosides, which we indeed observed in this study. However, the transcript levels of several proteins involved in the degradation of gangliosides, such as Neu1, HexA and Ctsa were also downregulated, whereas the mRNA for GM1a synthase (*B3GalT4*) was highly upregulated. These data point to alterations in the transcriptional regulation of this gene network in the *Cln3^Δex7/8^* cells. Interestingly, some of the genes involved in GSL degradation have been shown to be part of the CLEAR gene network (Coordinated Lysosomal Expression and Regulation) that is regulated by the Transcription Factor EB (TFEB) family [30,31]. Importantly, the *CLN3* gene is also part of this network, and activation of TFEB by oral administration of trehalose in the *Cln3^Δex7/8^* mouse model has been shown to ameliorate the neuropathological features [32]. Trehalose also resulted in a high degree of upregulation of the *Ctsa* and *Hexa* genes in JNCL fibroblasts [32]. Thus, further studies should be carried out to characterize the role of TFEB in the regulation of GSL biosynthetic genes, as it has not been studied if they are part of the CLEAR network.

The accumulation of GM3 in *Cln3^Δex7/8^* cells is most likely explained by the low level of GM2 synthase mRNA and protein. Many of the GSL synthesizing transferase enzymes reside in the Golgi where they form protein complexes with each other, which is thought to facilitate the direct passing of the products to the next glycosyltransferase in the biosynthetic pathway (Reviewed in [12,33]). These enzymes are actively transcriptionally regulated [15]. However, their levels and activity are also controlled by changes in the cellular localization of the complex, which in turn is influenced by the stoichiometry of the proteins within the complex. Thus, changes in the expression of one of the enzymes within the complex may result in altered localization and activity of several enzymes (Reviewed in [33]). For example, changes in the expression of GM2 synthase may alter the function of the transferases acting upstream or downstream of it.

Interestingly, although several human genetic disorders of ganglioside degradation are known, there are only few confirmed examples of gene defects in GSL biosynthesis. GM3 synthase deficiency leads to a very severe early-onset epilepsy, whereas GM2 synthase deficiency causes a form of hereditary spastic paraplegia [18,19]. A patient has been reported with an apparent GM2 synthase deficiency (genetically unconfirmed) with profound GM3 accumulation in brain and liver, associated with poor motor and physical development accompanied by frequent seizures and death by 3 months of age [34]. Due to the rarity of human diseases caused by GSL biosynthesis defects, most of the functional data have been derived from animal models. Knockout of GM2/GD2 synthase results in high levels of GM3 and GD3 gangliosides and lack of more complex gangliosides [35,36], and these mice show axonal degeneration and demyelination in the CNS. A double knockout mouse for GM2 and GD3 synthases has been generated, and these mice exhibit only GM3 ganglioside but lack the more complex gangliosides beyond this point [37]. Interestingly, these mice are prone to fatal audiogenic seizures and show an early degeneration of the peripheral nerves. In the future, it would be important to study in the JNCL mouse model if they show a similar pathology and if the pathological features can be linked to GM3 accumulation.

GM3 is highly abundant during early embryonic development of the brain. In later stages of development, the amount of GM3 decreases, although GM3 synthase is not differentially expressed during the embryonic development. It has been shown that the expression of GM2 synthase significantly increases during fetal development or differentiation of cultured primary neural precursors [38]. This results in a reduction in GM3 level and a concomitant increase in the more complex gangliosides during embryonic brain development. 

Studies on animal models have shown that the complex gangliosides are not required for early embryonic brain development, but they are essential for the maturation and maintenance of the brain later on, once developed. During post-natal brain maturation, both the levels and degree of sialylation of gangliosides increase [39,40]. In the adult brain, 75% of the sialic acid content is bound to gangliosides [41], and the main gangliosides of adult human brain are GM1a, GD1a, GD1b and GT1b [40] which share the same neutral glycan core but exhibit a varying number of sialic acids [41].

GM3 accumulation and reduction of the more complex gangliosides in JNCL patient brains might contribute to the pathogenesis of JNCL by impairing brain maturation or maintenance. However, these changes in GSL levels may also have more direct molecular consequences. GM1a and GD1a have been suggested to be essential for the myelination process in the brain [42], and GM1a displays neuroprotective properties by protecting cells from glutamate-induced excitotoxicity [43]. In addition, GM1a clusters in the cell membrane can activate TrkA nerve growth factor receptors in the absence of neurotrophins and induce neurite outgrowth via Lyn, Akt and MAPK signaling [44]. Furthermore, in cerebellar granule cells, GM1a associates with the neuronal cell adhesion molecule TAG-1, which is required for the correct localization of the potassium channels Kv1.1 and Kv1.2 [45,46]. These findings suggest that GM1a level in the brain is important for the survival of neurons and that reduced GM1a might indeed contribute to JNCL pathogenesis.

Recently, considerable attention has been paid to identify pathological features that may be common to neurodegenerative diseases, including Alzheimer’s disease (AD) and Parkinson’s Disease (PD), but also lysosomal storage disorders with neuropathology. Interestingly, CLN3 has been suggested to be a physiological regulator of the Alzheimer amyloid precursor protein metabolism [47]. Intriguingly, GM3 was shown to be elevated in AD and Lewy Body dementia, whereas GM1a levels were found to be decreased in patients with AD, PD and Huntington’s disease (HD) [48,49,50,51,52,53,54,55], suggesting that altered GM3 and/or GM1a levels might indeed be common to JNCL and some more common neurodegenerative diseases. In addition, GM1a has been shown to block the cytotoxicity of the amyloid β peptide and to be protective against other neuronal insults in cell culture models [43,56]. Thus, modulation of ganglioside levels may even exhibit therapeutic potential, as has been shown in the mouse model for AD, where upregulation of GM1a and GD1a ganglioside levels by elimination of GD3 synthase reduced the plaque load and resulted in improved memory [57]. Furthermore, restoration of GM1a level by exogenous administration of GM1a in an HD cell model protected the cells from apoptosis by activating the phosphatidylinositol 3-kinase/protein kinase B pathway [49]. Exogenous GM1a supplementation has been shown to be relatively safe, apart from a few cases exhibiting peripheral neuropathy due to presence of anti-GM1a antibodies [58]. Importantly, at least a small fraction of gangliosides or their precursors can cross both the blood-brain barrier and the placenta [59]. However, the blood-brain barrier has been shown to be leaky in the JNCL mouse model, which might facilitate the treatment of JNCL by GM1 substitution [60,61,62].

In summary, we here describe hitherto undescribed alterations in the amounts of a-series gangliosides in the cerebellar neuronal precursor cells of the *Cln3^Δex7/8^* mouse model. Furthermore, we could show that the transcript amounts of proteins involved in GSL synthesis and degradation were altered. These data point to aberrations in the transcriptional regulation of the respective genes as a potential cause for the altered ganglioside amounts. Since GM3 ganglioside accumulation is per se pathogenic and may contribute to JNCL pathogenesis, it might be possible to revert the GM3 accumulation by affecting the transcription of the genes involved in the regulation of its synthesis, especially GM2 synthase/*B4GalNT1* gene. In future studies, it will be important to characterize the regulation of the ganglioside synthesis/degradation gene network in more detail and to identify transcription factors that are responsible for their regulation. In addition, possible alterations in signal transduction in JNCL cell should be characterized, since signaling processes are highly important for transcriptional regulation. This knowledge might help to identify novel or even approved drugs that target the pathways important for the regulation of ganglioside processing proteins and might thus provide novel therapeutic alternatives for JNCL.

## 4. Materials and Methods

### 4.1. Cell Culture

The experiments were carried out on conditionally immortalized cerebellar (Cb) granule neuronal precursor cells, derived from P4 *Cln3*^+/+^ and homozygous *Cln3^Δex7/8^* CD1 mice [2]. The *Cln3^Δex7/8^* mutation present in these cells corresponds to the 1.02 kb deletion found in human JNCL patients. Culture of these cells was done as described by Fossale et al. [2]. Briefly, the cells were maintained in Dulbecco’s Modified Eagle’s Medium (DMEM) containing 4.5 g/L glucose, 110 mg/L sodium pyruvate and l-glutamate (all from Gibco, Thermo Fisher Scientific, Karlsruhe, Germany) supplemented with 10% fetal bovine serum, 100 μg/mL Penicillin/Streptomycin (Invitrogen, Thermo Fisher Scientific, Karlsruhe, Germany), 24 mM potassium chloride and 200 μg/ml Geneticin G418 (Invitrogen), at 33 °C under 5% or 8% CO_2_.

### 4.2. Antibodies and Toxins; Immunostaining

CTX-FITC and CTX-A488 (Alexa Fluor 488) were from Invitrogen. Anti-GM2 synthase polyclonal rabbit antibody (Cat. AV45116) was from Sigma-Aldrich (Taufkirchen, Germany), the monoclonal anti-GAPDH and the polyclonal LAMP-1 antibody from Abcam (Cambridge, UK). Anti-GD1a antibody (mouse monoclonal MAB5606) was from Millipore (Darmstadt, Germany), anti-GM1 antibody (rabbit, Cat. 345757) and anti-GM2 antibody (rabbit, Cat. 345759) were from Calbiochem (Darmstadt, Germany). Either A488- or A546-conjugated donkey anti-rabbit antibody (1:300; Invitrogen) or A488-conjugated donkey anti-mouse antibody (Invitrogen) were used as secondary antibodies for immunostaining.

The cells were grown on coverslips for one day, washed once with PBS (phosphate buffered saline) prior to fixation for 20 min with 4% paraformaldehyde (PFA). After washing with PBS, a quenching step using 50 µM glycine in PBS was carried out prior to three washing cycles with PBS. Afterwards, the cells were blocked and permeabilized with 1% BSA and 50 µg/mL digitonin in PBS for 20 min at room temperature, followed by an incubation with the respective primary antibody for one hour at room temperature. The cells were washed with PBS three times for five minutes each. Subsequently, incubation with the fluorochrome-coupled secondary antibody for one hour at room temperature was performed. After washing, the coverslips were embedded in a mounting medium containing DAPI (4′,6′-diamidino-2-phenylindole, (Sigma-Aldrich). The fluorescence images have in some cases as a whole been subjected to contrast or brightness adjustments. No other manipulations have been performed unless otherwise stated (see Figure 4). Samples were analyzed with a Zeiss LSM710 Confocal Laser Scanning Microscope (Carl Zeiss, Oberkochen, Germany).

### 4.3. RNA Isolation, cDNA Synthesis and Quantitative Real-Time PCR

For RNA isolation, the cells were suspended in Trizol reagent (Invitrogen), allowed to stand for 5 min at room temperature (RT) before addition of 200 µL chloroform. The samples were mixed and allowed to rest for 5 min at RT, followed by centrifugation at 12,000 *g* for 15 min at 4 °C. The supernatant (upper clear phase) was transferred to a new tube, 400 µL isopropanol was added and the tubes were inverted several times before another rest at RT for 10 min. Subsequently, the samples were centrifuged at 12,000 *g* for 10 min at 4 °C. The supernatant was discarded, the pellet washed twice with 75% ethanol. The pellet was air dried before dissolving in 25 µL DEPC (diethylpyrocarbonate, Sigma-Aldrich) water. Subsequently, the RNA concentrations were measured. 

For qPCR, 3 µg of total RNA was reverse-transcribed using the M-MuLV reverse transcriptase (NEB, Frankfurt am Main, Germany), the M-MuLV buffer and 150 fmol oligo(dT) primers in a total volume of 45 µL. qRT-PCR was performed using the CFX Connect Real-Time OCR Detection System (Bio-Rad, Munich, Germany). Annealing temperature was 60 °C for all primers, the sequences of which are shown in Table 3. Samples were processed as duplicates using 0.2 µL of 10-fold diluted cDNA in a final volume of 10 µL with iTaqTMUniversal SYBR Green Supermix (Bio-Rad). The ∆Ct-method was used to quantify the PCR products. The geometric mean of the reference genes RPL13a and B2M was used for normalization.

### 4.4. Western Blot and Dot Blot Analysis

Cells were harvested in lysis buffer (50 mM Tris pH 7.4; 150 mM NaCl; 2 mM EDTA; 1% NP-40) supplemented with protease inhibitor cocktail (Sigma-Aldrich) and incubated on ice for 30 min. The cell lysate was cleared by centrifugation, and the protein amount was measured using Bradford assay. Equal protein amounts were analyzed by 10% SDS polyacrylamide gel electrophoresis and Western blot. For immunoblotting, the membranes were blocked with 5% non-fat dried milk in TBST (10 mM Tris, 150 mM NaCl, 0.05% Tween 20) and incubated with the respective primary antibody overnight at 4 °C. After washing, the blots were incubated with HRP-conjugated secondary antibody in TBST for 1 h at room temperature. After washing, detection of the signal was carried out using enhanced chemiluminescence.

Proliferating WT and *Cln3^Δex7/8^* cells were seeded on 6 well plates to a confluence of 20–30%, grown up to 80% density, and lysed with CellLytic M cell lysis reagent (Sigma-Aldrich) containing protease inhibitor cocktail. Protein amounts were determined using Bradford assay, and dots with equal total protein amount were applied on nitrocellulose membranes. The membranes were blocked with 5% milk powder in PBS. CTX-HRP (Invitrogen, C34780) was used to detect GM1, and the ECL reaction imaged using Intas ChemoStar (Intas, Göttingen, Germany). Intensity analysis was performed with ImageJ (National Institutes of Health, Bethesda, MD, USA) after background correction. Thereby, the mean background value was subtracted from all intensity values, and all values were normalized to the mean signal intensity value of the WT cells. Statistical significance was determined with 2-way Anova.

### 4.5. Flow Cytometry and CTX-FITC Fluorometry

For flow cytometry, 4 × 10^5^ proliferating WT and Cln3*^Δex7/8^* cells were seeded per well on 6 well plates. After 24 h, the cells were trypsinized and resuspended into ice-cold PBS containing 20 mM HEPES. Subsequently, the cells were either permeabilized with 11 µg/mL digitonin (for intracellular labeling) or left non-permeabilized (for cell surface labeling), and incubated with 1 µg/mL CTX-FITC. Labeled cells and unlabeled autofluorescence controls were measured with a BD FACSAria flow cytometer (10,000 events per sample). WinMDI 2.9 software (J. Trotter, Purdue University, West Lafayette, IN, USA) was used to compute the geometrical means which were corrected for autofluorescence and normalized pairwise to the WT sample to adjust for staining and treatment variations. Paired *t*-test was used to test for statistical significance.

For fluorometry, 1.0 × 10^5^ proliferating WT and Cln3*^Δex7/8^* cells per well were seeded in 24 well plates. After 24 h in culture, the cells were washed twice with PBS and fixed with 4% PFA. Cells were either left non-permeabilized (surface staining) or permeabilized (total staining) with 20 μg/mL digitonin and labeled with CTX-FITC (1 µg/mL). Fluorescence signal was measured with a PerkinElmer Victor X3 plate reader (PerkinElmer, Rodgau, Germany), with each treatment group represented at least as triplicate. Arithmetic mean signals were corrected for autofluorescence. Unpaired Student’s *t*-test was used to test for statistical significance.

### 4.6. High-Performance Liquid Chromatography of Gangliosides

Glycosphingolipids were analyzed essentially as described by Neville et al. [63]. Lipids from cells were extracted with chloroform:methanol (1:2, *v*/*v*) overnight at 4 °C. GSLs were then further purified using solid phase C18 columns (Telos, Kinesis, UK). After elution, GSLs were dried under a stream of nitrogen and treated with ceramide glycanase (prepared in house from the medicinal leech Hirudo medicinalis/verbena) to obtain oligosaccharides from GSLs. Liberated glycans were then fluorescently-labelled with anthranillic acid (2AA). Excess 2AA label was removed using DPA-6S SPE columns (Supelco, PA, USA). Purified 2AA-labelled oligosaccharides were separated and quantified by normal phase high-performance liquid chromatography (NP-HPLC) as previously described [63]. The NP-HPLC system consisted of a Waters Alliance 2695 separations module and an in-line Waters 2475 multi λ-fluorescence detector set at Ex λ360 nm and Em λ425 nm. The solid phase used was a 4.6 × 250mm TSK gel-Amide 80 column (Anachem, Luton, UK). A standard 2AA-labelled glucose homopolymer ladder (Ludger, Oxfordshire, UK) was included to determine the glucose units (GU) of the HPLC peaks. Individual GSL species were identified by their GU values and quantified by comparison of integrated peak areas with a known amount of 2AA-labelled BioQuant chitotriose standard (Ludger). Results were normalized to protein content.

### 4.7. Statistical Analysis

All experiments were performed at least three times unless otherwise stated. For information on the statistical tests used, please refer to the figure legends and the sections of materials and methods describing the respective methods. Values of *p* < 0.05 were considered significant (*) while values of *p* < 0.01 were considered very significant (**) and *p* < 0.001 extremely significant (***).

## Figures and Tables

**Table 1:** Genes and enzymes involved in a-series ganglioside biosynthesis. Only steps beyond LacCer are shown.

Gene	Enzyme	Alternative Names	Synthesis Step
*ST3Gal5*	Lactosylceramide sialyltransferase	GM3 synthase, Sial-T1	LacCer to GM3
*B4GalNT1*	GM3 *N*-acetyl-galactosaminyl transferase	GM2 synthase, GalNAc-T, GM3 sialyltransferase	GM3 to GM2
*B3GalT-4*	GM2 galactosyltransferase	GM1 synthase, Gal-T2	GM2 to GM1
*ST3Gal2*	GM1 sialyltransferase	GD1a synthase, Sial-T4	GM1 to GD1a
*ST3Gal3*	GD1a sialyltransferase	GT1a synthase, Sial-T5	GD1a to GT1a

**Table 2:** Quantitative real-time PCR analysis of the transcript levels of genes involved in ganglioside synthesis and degradation in mouse cerebellar precursor cells. Data are a summary of three independent experiments.

Gene Symbol	Enzyme/Protein (Function)	Mean Expression Level ± SD		*p*-Value
		WT	*Cln3^Δex7/8^*	
***St3gal2***	GM1 sialyltransferase	1.0 ± 0.01	1.02 ± 0.01	0.939
***B4galNT1***	β-1,4-*N*-acetyl-galactosaminyl transferase 1	1.0 ± 0.03	0.12 ± 0.01	4 × 10^−4^
***Neu1***	neuraminidase 1 (GD1a to GM1)	1.0 ± 0.07	0.50 ± 0.04	0.036
***Glb1***	β-galactosidase (GM1 to GM2)	1.0 ± 0.002	0.57 ± 0.006	0.060
***Gm2a***	GM2 activator protein (assists in degradation of GM1 and GM2)	1.0 ± 0.01	0.78 ± 0.01	0.474
***Ctsa***	cathepsin A (assists in degradation of GD1a and GM1a)	1.0 ± 0.03	0.29 ± 0.04	0.005
***Hexa***	hexosaminidase A (GM2 to GM3)	1.0 ± 0.03	0.20 ± 0.04	0.007
***B3galT4***	β-1,3-galactosyltransferase, polypeptide 4 (GM2 to GM1)	1.0 ± 0.09	4.46 ± 0.52	0.056

**Table 3:** Oligonucleotides used for quantitative real-time PCR analysis.

Target Gene	Primer Name	Sequence 5′-3′
***St3Gal2***	St3Gal2-fwd	GACGCCAGCACCTCTGAATGGT
	St3Gal2-rev	TTGTAGCATCATCCACCACCGC
***B4GalNT1***	B4GalNT1-fwd	GAGATATACCAGGTGAACCTGAG
	B4GalNT1-rev	CTGCCGGTTGAGTTTATCCA
***Neu1***	Neu1-fwd	CACCCTGAGTTCCGAGTGAACC
	Neu1-rev	GCTGCTTCTTTCCATCCGTGCT
***Glb1***	Glb1-fwd	CGGTCGACCTCCAATTCTTCGG
	Glb1-rev	TACTTGACCCTTGGACCACCCA
***Gm2a***	Gm2a-fwd	AGCCTCACGATCCAACCTGACC
	Gm2a-rev	GAGCTCCACCTTCTGAGGAGCA
***Ctsa***	Ctsa-fwd	TCACCATCAAGGGTGCCGGA
	Ctsa-rev	GGATTTCCATGGGTTGCAGCGG
***Hexa***	Hexa-fwd	CTACATCCAGACGCTGCTGGAC
	Hexa-rev	TACTGGCATTTCTTCCCGCCAC
***B3GalT4***	B3GalT4-fwd	GGTTAAAGCCGTCCTCCCACCT
	B3GalT4-rev	ACCAGTAGTAGGACCGCCAGGA
***RPL13a***	RPL13a-fwd	GTTCGGCTGAAGCCTACCAG
	RPL13a-rev	TTCCGTAACCTCAAGATCTGCT
***B2M***	B2M-fwd	CCTGGCTCACACTGAATTCACC
	B2M-rev	ATGTCTCGATCCCAGTAGACGG

## Abbreviations

2AA	Anthranillic acid
AD	Alzheimer’s disease
Akt	RAC-α serine/threonine-protein kinase
BSA	Bovine serum albumin
Cb	Cerebellar
CLEAR	Coordinated Lysosomal Expression and Regulation
CLN3	Ceroid lipofuscinosis, neuronal
CNS	Central nervous system
CTX	Cholera toxin subunit B
DEPC	Diethylpyrocarbonate
ECL	Enhanced chemiluminescence
ER	Endoplasmic reticulum
FACS	Fluorescence-activated cell sorting
FITC	Fluorescein isothiocyanate
GalCer	Galactosylceramide
GSL	Glycosphingolipid
GU	Glucose units
HD	Huntington’s disease
HPLC	High performance liquid chromatography
JNCL	Juvenile neuronal ceroid lipofuscinosis
LacCer	Lactosylceramide
LAMP-1	Lysosomal-associated membrane protein 1
MAPK	Mitogen-activated protein kinase
NP-HPLC	Normal phase high-performance liquid chromatography
PBS	Phosphate buffered saline
PD	Parkinson’s disease
qRT-PCR	quantitative real-time polymerase chain reaction
SDS	Sodium dodecyl sulfate
SPE	Solid Phase Extraction
TFEB	Transcription Factor EB
TrkA	Tropomyosin receptor kinase A
